# Reappraising the Role of Allogeneic Hematopoietic Stem Cell Transplantation in Relapsed and Refractory Hodgkin’s Lymphoma: Recent Advances and Outcomes

**DOI:** 10.3390/jpm12020125

**Published:** 2022-01-18

**Authors:** Taha Al-Juhaishi, Azra Borogovac, Sami Ibrahimi, Matthew Wieduwilt, Sairah Ahmed

**Affiliations:** 1 Stephenson Cancer Center, University of Oklahoma, Oklahoma City, OK 73104, USA; Azra-Borogovac@ouhsc.edu (A.B.); Sami-Ibrahimi@ouhsc.edu (S.I.); Matthew-Wieduwilt@ouhsc.edu (M.W.); 2 MD Anderson Cancer Center, University of Texas, Houston, TX 77030, USA; sahmed3@mdanderson.org

**Keywords:** Hodgkin’s lymphoma, high-dose chemotherapy, autologous hematopoietic stem cell transplant, allogeneic hematopoietic stem cell transplant

## Abstract

Hodgkin’s lymphoma is a rare yet highly curable disease in the majority of patients treated with modern chemotherapy regimens. For patients who fail to respond to or relapse after initial systemic therapies, treatment with high-dose chemotherapy and autologous hematopoietic stem cell transplantation can provide a cure for many with chemotherapy-responsive lymphoma. Patients who relapse after autologous transplant or those with chemorefractory disease have poor prognosis and represent a high unmet need. Allogeneic hematopoietic stem cell transplantation provides a proven curative therapy for these patients and should be considered, especially in young and medically fit patients. The use of newer agents in this disease such as brentuximab vedotin and immune checkpoint inhibitors can help bring more patients to transplantation and should be considered as well.

## 1. Introduction

Hodgkin’s lymphoma (HL) is a rare lymphoma of B-cell origin which consists of two subtypes: classical Hodgkin’s lymphoma (cHL; around 90% of all HL cases), which is further subdivided based on histological findings into nodular sclerosis, mixed cellularity, lymphocyte-rich, and lymphocyte depleted variants; and nodular lymphocyte-predominant Hodgkin lymphoma (up to 10% of all HL cases) [1]. HL affects 8000–9000 patients in the US per year, and the more common cHL subtype is considered as a highly curable B-cell malignancy when treated with combination chemotherapy, with or without the addition of radiotherapy [2]. It is estimated that up to 10% of HL cases are refractory to initial therapy, and up to 30% relapse after initial response; however, approximately half of these patients can still be cured using high-dose chemotherapy and autologous hematopoietic stem cell transplant (AHSCT) [2,3,4,5]. For those who are not candidates for AHSCT or whose disease relapses afterwards, the prognosis is grim. Despite the success of the antibody–drug conjugate brentuximab vedotin and immune checkpoint inhibitors in the treatment of relapsed and refractory disease, their curative potential is debatable and the majority of patients eventually progress [6]. 

Allogeneic hematopoietic stem cell transplantation (AlloHSCT) is a known curative therapeutic option for a variety of hematologic and immunologic conditions. More than a decade of progress has made this modality safer and accessible to more patients worldwide [7]. The benefits of AlloHSCT go beyond the anticancer effects of high-dose chemotherapy and radiotherapy, to the more durable graft-versus-lymphoma phenomenon [8,9,10]. Furthermore, the recognition of the benefits of graft-versus-lymphoma effects has allowed clinicians to use lower doses of chemo- or radiotherapy (reduced-intensity conditioning, RIC) with a reduction in toxicity while achieving good outcomes. This in turn has decreased the morbidity and mortality of AlloHSCT and permitted its use in older, less fit and heavily pre-treated patients [11]. In this review, we will discuss the development and evidence of AlloHSCT in the treatment of cHL including our approach to utilizing this potentially curative therapy. Due to the differences in disease biology and treatment approaches in nodular lymphocyte-predominant HL, this review will not address that subtype and only focus on classical Hodgkin’s lymphoma (cHL). 

## 2. Disease Failure after Autologous Stem Cell Transplant

Approximately up to 10% of early-stage (non-bulky stage I or II by the Ann Arbor system) disease and about 20–30% of advanced-stage disease treated with modern chemotherapy regimens can be refractory or relapse after initial response [12]. Despite all the progress in the field, the standard-of-care-therapy approach remains centered around using high-dose chemotherapy and AHSCT in patients with lymphoma that is responsive to chemotherapy (referred to as chemosensitive disease) [13]. This approach can potentially cure close to 50% of these patients, as shown in several randomized trials and a meta-analysis that clearly described the benefits of AHSCT in this setting [4,5,14,15,16,17,18]. Several studies have tried to identify the disease factors that could predict relapse after AHSCT [19,20]. One important predictor of relapse in these studies was the duration of lymphoma remission after initial chemotherapy, wherein relapse within 12 months of initial treatment was associated with a higher risk of relapse. Other risk factors included primary refractory disease, extranodal disease, bulky disease, active disease at the time of transplant and the presence of B symptoms from lymphoma. Identification of these high-risk features allowed clinical investigators to develop protocols for post-transplant maintenance therapies to address this unmet need. 

One important and successful example of this was the international randomized phase 3 AETHERA study [21]. This study randomized high-risk HL patients post-transplant, who would receive either a placebo or brentuximab vedotin for a total of up to 16 cycles. Brentuximab vedotin (BV) is an antibody–drug conjugate that targets CD30 on the surface of the malignant Reed–Sternberg cell; the antibody is linked to a microtubule inhibitor called monomethyl auristatin E. The binding of BV to CD30 on the tumor cell membrane triggers a cascade of events that ultimately results in apoptotic death of the CD30-expressing cell. Prior to the AETHERA study, BV was already shown to be effective in treatment of relapsed/refractory cHL, both as a single agent and in combination with other chemotherapy agents [22]. High-risk patients were defined as having disease refractory to frontline therapy, progression within 12 months from the end of frontline therapy, or having extranodal relapsed disease. This maintenance approach led to improvement in 5-year progression-free survival (PFS) from 41% with a placebo to 59% with BV, with a hazard ratio (HR) of 0.521. No benefit in overall survival (OS) has been reported yet. Of note, more patients in the placebo arm underwent AlloHSCT (23 vs. 12). These results led to the approval of BV maintenance indication by the US Food and Drug Administration (FDA) in 2015. A recent small phase 2 study explored the addition of checkpoint inhibitor nivolumab to BV while shortening the maintenance treatment duration to only eight cycles. The study enrolled 59 high-risk patients with criteria similar to that of AETHERA. The 18-month PFS and OS were reported to be 95% and 98%, respectively, as only one patient relapsed. Toxicities were consistent with the known effects of these agents, with neuropathy and neutropenia being the most common in this case [23]. 

## 3. Role of Allogeneic HSCT in Relapsed/Refractory HL

The prognosis of patients who relapse after AHSCT is poor. Studies have shown that the median survival for these patients is around 2.4 years [24,25]. Outcomes seem to have improved over the last three decades but a shorter time to relapse post-AHSCT was associated with worse median survival post-progression (median post progression survival of about 1 year for those who relapsed within 12 months of AHSCT) [25]. The loss of life and loss to society are significant as many of these patients are young adults. A select group of these patients with relapsed and refractory HL can benefit from the curative potential of AlloHSCT, as shown in studies dating back to the 1980s [26]. Similar to the graft-versus-leukemia effects seen in myeloid neoplasms, a graft-versus-lymphoma (GVL) effect was described by several groups [8,27,28]. The evidence for GVL in cHL comes from several indirect observations. These include induction of remission by reducing immunosuppression post-transplant, or by using donor lymphocyte infusion. Moreover, the fact that reduced-intensity conditioning transplants can induce remissions in relapsed or refractory disease is a good illustration of the strength of GVL. AlloHSCT is also associated with significant toxicities, including graft-versus-host disease (GVHD), which could increase non-relapse mortality risk. GVHD, and especially the chronic GVHD, has been shown to be associated with lower risk of relapse, which may reflect the benefits of graft-mediated immune surveillance in the prevention of lymphoma relapse [10,29]. Given the lack of randomized prospective trials evaluating the role of AlloHSCT in cHL, multiple retrospective, small prospective phase 2 and registry studies have shown the benefit of this procedure in selected fit patients with relapsed disease [30]. Nevertheless, the optimal timing of AlloHSCT and appropriate group of patients remain an open debate between experts in the field and many have advocated to reserve this therapy for patients that relapse post-AHSCT [31,32]. One of the largest phase 2 studies of AlloHSCT in cHL was conducted by the Spanish lymphoma group (GELTAMO) and the European Society for Blood and Marrow Transplantation (EBMT). The study included 78 patients with relapsed cHL and showed 1-year and 4-year PFS of 48% and 24%, respectively. The 1-year and 4-year OS were 71% and 43%, respectively, and NRM was 17% at 2 years. Chronic GVHD was associated with lower risk of relapse again, indicating the benefits of a GVL effect [33]. A summary of the other key studies that evaluated the role of AlloHSCT in cHL is shown in Table 1. 

## 4. Role of the Conditioning Regimen

Myeloablative conditioning (MAC) has historically been the preferred conditioning intensity for aggressive hematologic malignancies. This is partially related to the idea of overcoming chemoresistance by increasing the total systemic dose to the highest possible amount while avoiding significant damage to organs other than the marrow. This practice in turn restricted the utilization of AlloHSCT to fit and young patients, as high morbidity and mortality are associated with this approach. The development of reduced intensity conditioning (RIC) is a major landmark in the progress of the transplant field. It expanded the eligibility to many more patients and highlighted the anticancer properties of a healthy donor immune system [36,41]

A study using the international bone marrow transplant registry (IBMTR), which included 114 patients with lymphoma who received MAC AlloHSCT, showed 3-year transplant-related mortality (TRM) of 22%. The rate of disease progression at 3 years was noted to be 52%. There were no differences seen in TRM, PFS and OS between HL and the other lymphoma types [42]. As alluded to before, the study by the Spanish group and the EBMT, which included 78 patients with cHL treated with mostly fludarabine and melphalan reduced intensity conditioning followed by matched related or unrelated AlloHSCT, showed 1-year non-relapse mortality (NRM) of 15%. The 4-year PFS and OS were 24% and 43%. Being in remission at the time of transplant and development of chronic GVHD were associated with lower risk of relapse [33].

Overall, these studies highlight a common theme in the field of hematopoietic stem cell transplantation, which is balancing the anticancer benefits of MAC against the increased risk of associated non-relapse mortality. The role of maintenance therapies in cHL post-AlloHSCT remains to be investigated. One potential area that is worth exploring is the role of maintenance therapies after RIC AlloHSCT and whether this strategy could help decrease the risks of relapse and NRM, especially for those with high-risk and active disease at the time of transplant. 

## 5. Role of Alternative Donor HSCT in Relapsed or Refractory cHL

The rise of alternative donors, such as haploidentical AlloHSCT donors and umbilical cord units, have further expanded the availability of transplantation to additional groups of patients [43,44]. Minorities and people in developing countries are particularly underrepresented in, or have no access to, national donor registries. The EBMT and Eurocord investigated the benefits of umbilical cord blood (UCB) transplants in a large registry study of 131 adult patients with HL. A total of 117 (89%) of the patients were able to engraft with a median time of 18 days (range, 6–61). The 4-year relapse rate, NRM, PFS and OS were 44%, 31%, 26%, and 46%, respectively. Having residual disease at the time of transplant was associated with worse outcomes, while receiving RIC regimen of cyclophosphamide, fludarabine, and low dose total body irradiation was associated with better PFS and OS in multivariate analysis [45].

In terms of haploidentical transplants, the advent of the use of post-transplant cyclophosphamide in the prevention of GVHD has made this modality an acceptable standard of care therapy for many hematologic diseases [46]. Several retrospective studies and at least one systematic review have suggested better PFS with haploidentical AlloHSCT compared to HLA-matched transplants in cHL [41,47,48,49]. This approach was further evaluated by larger registry-based studies using both CIBMTR and EBMT data. In the EBMT study, they compared patients who received haploidentical AlloHSCT with those that had matched sibling or unrelated donor transplants. A total of 90% of the patients in the haploidentical AlloHSCT group received RIC regimens and 100% had post-transplant cyclophosphamide, as expected. The composite endpoint of extensive-GVHD and relapse-free survival was better with haploidentical AlloHSCT compared to a matched-sibling donor (MSD), but similar compared to a matched-unrelated donor (MUD) (40% for haploidentical, 28% for MSD, and 38% for MUD). The 2-year relapse rate, PFS and OS were 39%, 43%, and 67%, respectively. Multivariate analysis showed haploidentical AlloHSCT to have a lower relapse rate compared to MSD, but one that is not significantly different from MUD transplants [50]. In the CIBMTR study, investigators compared 139 patients receiving haploidentical AlloHSCT to 457 others that had MSD transplants. All haploidentical AlloHSCT patients received RIC regimens and post-transplant cyclophosphamide for GVHD prophylaxis. They found no significant differences in PFS or OS; however, haploidentical AlloHSCT had lower risk of relapse. Haploidentical AlloHSCT also had a higher risk of acute GVHD, but a lower risk of chronic GVHD. The 3-year relapse rate, NRM, PFS and OS were 45%, 22%, 33%, and 49%, respectively [51]. Another recent small retrospective study evaluated the outcomes of haploidentical AlloHSCT in patients with active refractory cHL who received a salvage transplant with fludarabine and melphalan-based conditioning. This study included 8 patients treated at MD Anderson Cancer Center in the US and 7 patients treated at Fundeni Clinical Institute in Romania. They reported an overall response rate of 100%, with a 5-year PFS of 42.6% and a 5-year OS of 60.9%. The 5-year NRM was found to be 21.3%, but with 0% treatment-related mortality (one patient died of lung cancer more than 50 months after transplant). All relapses happened within the first year of transplant. This study, albeit small, shows very encouraging data in a difficult-to-treat group of patients [52]. 

The data discussed above show that alternative donor transplants are feasible and can provide durable benefits similar to sibling or matched unrelated donors. Of interest is the lower risk of relapse noted with haploidentical AlloHSCT in cHL. However, due to retrospective nature of all of these studies, this finding remains hypothesis generating and warrants validation in prospective trials. 

## 6. Checkpoint Inhibitors and Graft-Versus-Host Disease

Immune checkpoint inhibitors are one of the most successful types of therapies in modern oncology, especially in solid tumors [53,54,55]. They have shown to be active in relapsed and refractory cHL in multiple prospective studies, and the FDA has approved two PD-1 inhibitors, pembrolizumab and nivolumab, for these indications [56]. Several reports have suggested that AlloHSCT in the setting of prior exposure to PD-1 inhibition may be associated with higher than normal rates of early transplant-related complications, of which the most important was severe acute GVHD. [57,58,59]. These results prompted a warning from the FDA to use caution using PD-1 blockade as a bridge to AlloHSCT. A systematic review of 122 patients who had received AlloHSCT after checkpoint inhibitors compared to a control group of 978 patients showed higher risk of grade III/IV acute GVHD (28% vs. 8%, *p* = 0.02) but similar rates of chronic GVHD (26% vs. 29%, *p* = 0.82). Non-relapse mortality was similar between the two groups (15% vs. 19%, *p* = 0.35) [60]. Another study evaluated the effects of checkpoint inhibitors before and after AlloHSCT in patients with hematologic malignancy; the 107 patients that received these drugs prior to transplant and the 176 that received them afterwards were included in this analysis. cHL was the most common disease in both groups. In the group that received checkpoint inhibitors before AlloHSCT, they reported rates of acute GVHD, chronic GVHD, and GVHD-related mortality to be 56%, 29% and 11%, respectively. In the group that received checkpoint inhibitors after AlloHSCT, these rates were 14%, 9%, and 7%, respectively [61]. A recent international retrospective study of 209 patients with cHL who underwent AlloHSCT after PD-1 inhibitors showed similar results with grade III/IV acute GVHD of 15%, 2-year chronic GVHD of 34%, and 2-year NRM of 14%. Interestingly, the study showed that longer duration between PD-1 exposure and AlloHSCT and the use of post-transplant cyclophosphamide were both associated with better outcomes [62]. Collectively, these data suggest higher risk of complications when immune checkpoint inhibitors are used in the peri-transplant setting and therefore additional mitigation strategies are needed for safer delivery of AlloHSCT to these patients. The use of post-transplant cyclophosphamide for GVHD prophylaxis in this setting is of interest and warrants further investigation. 

## 7. Authors’ Approach to AlloHSCT in HL

We recommend high-dose myeloablative chemotherapy followed by autologous stem cell rescue as standard second (or beyond second if not attempted before) line of therapy for patients with relapsed chemosensitive disease. We prefer autologous over allogeneic stem cell transplants for patients with no prior history of transplants due to lower associated toxicities. For those with disease refractory to chemotherapy, we recommend incorporation of brentuximab vedotin or checkpoint inhibitor-based regimens, then proceeding to autologous stem cell transplant, preferably in a positron emission tomography (PET) negative complete remission if able. If the disease is refractory to approved agents, then we recommend clinical trials with investigational agents if available. If no clinical trials are available, then additional attempts of chemotherapy incorporating other active chemotherapy agents or radiation could be considered. Responses in the settings are usually transient and thus consolidation with allogeneic stem cell transplantation is highly recommended. We recognize the potentially lower risk of relapse with haploidentical donor transplants compared to other donors, however due to the retrospective nature of the data and lack of clear survival benefit, we do not preferentially recommend haploidentical donors over HLA-matched donor sources. We recommend reduced-intensity conditioning regimens, such as the combination of fludarabine and melphalan, for most patients due to lower risks of transplant-associated mortality. We recommend using a post-transplant cyclophosphamide approach in patients with heavy exposure to immune checkpoint inhibitors. We do also advise a few weeks’ wash-out period before transplant for patients on checkpoint inhibitor therapy, while acknowledging the lack of strong data to support this recommendation. For patients with relapsed disease after AlloHSCT or those unable to receive one, we recommend clinical trials with promising investigational therapies, which also include CD30-directed chimeric antigen receptor therapy and other cellular therapy modalities such as the combination of NK-cell therapy with AFM13 monoclonal antibodies [63,64].

## 8. Conclusions

While cHL is a rare and generally curable lymphoma for the majority of patients, there remains a gap in highly efficacious therapy for patients that experience relapsed or refractory disease. Despite the tremendous progress made in understanding this disease, high-dose chemotherapy and autologous stem cell transplant remain the most proven curative therapy for patients whose disease responds to salvage systemic therapies. Relapses after autologous stem transplant should be treated on clinical trials when possible, or with strong consideration for consolidation with reduced intensity conditioning allogeneic stem cell transplantation, using the best available donor. The role of haploidentical transplant in prevention of relapse compared to other types of donors is worth further investigation in prospective randomized trials. 

## Figures and Tables

**Table 1:** Summary of the key studies evaluating the role of AlloHSCT in cHL.

Study	Type	Number of Patients	Prior AHSCT	Donor Type	Conditioning	PFS	OS
Sureda et al. [34]	Retrospective registry (EBMT)	168	52%	MSD for more than 70%, rest are MUD	MAC 47%, RIC 53%	20% MAC and 18% RIC at 5 years	22% MAC and 28% RIC at 5 years
Anderlini et al. [35]	Single center prospective	58	83%	MSD 43%, 57% MUD	RIC 100% (fludarabine and Melphalan)	32% at 2 years	64% at 2 years
Robinson et al. [36]	Retrospective registry (EBMT)	285	80%	MSD 60%, MUD 33%	RIC 100% Fludarabine based (79.5%), low dose TBI (16%)	25% at 3 years	29% at 3 years
Devetten et al. [37]	Retrospective registry (CIBMTR)	143	89%	Unrelated 100% (matched in 77%)	RIC/NMA 100% Melphalan based 34%	20% at 2 years	37% at 2 years
Marcais et al. [38]	Multicenter retrospective in France	191	92%	MSD 60%, MUD 40%	RIC 100% Fludarabine and busulfan in 36%	39% at 3 years	63% at 3 years
Kako et al. [39]	Retrospective registry (Japanese society for HSCT)	122	67%	MSD 39% MUD 17%	MAC 30% RIC 62%	31%	66% at 3 years
Sarina et al. [40]	Retrospective multicenter in Italy	104	100%	MSD 55% MUD 32%	RIC 100% (Fludarabine based in 100%)	31% at 2 years	57% at 2 years

MSD = matched sibling donor, MUD = matched unrelated donor, MAC = myeloablative conditioning, RIC = reduced-intensity conditioning, NMA = non-myeloablative conditioning.

## Data Availability

Not applicable.
